# Single-cell RNA sequencing reveals the cellular and molecular changes that contribute to the progression of lung adenocarcinoma

**DOI:** 10.3389/fcell.2022.927300

**Published:** 2022-08-15

**Authors:** Bing Liu, Chen Wang, Zhanjie Fang, Jing Bai, Ying Qian, Yuanyuan Ma, Xiuyan Ruan, Shi Yan, Shaolei Li, Yaqi Wang, Bin Dong, Xin Yang, Meng Li, Xuefeng Xia, Hongzhu Qu, Xiangdong Fang, Nan Wu

**Affiliations:** ^1^ Key Laboratory of Carcinogenesis and Translational Research (Ministry of Education), Department of Thoracic Surgery II, Peking University Cancer Hospital & Institute, Beijing, China; ^2^ CAS Key Laboratory of Genome Sciences and Information, Beijing Key Laboratory of Genome and Precision Medicine Technologies, Beijing Institute of Genomics, Chinese Academy of Sciences/China National Center for Bioinformation, Beijing, China; ^3^ University of Chinese Academy of Sciences, Beijing, China; ^4^ Geneplus-Beijing Institution, Peking University Medical Industrial Park, Zhongguancun Life Science Park, Beijing, China; ^5^ Key Laboratory of Carcinogenesis and Translational Research (Ministry of Education), Central Laboratory, Peking University Cancer Hospital and Institute, Beijing, China; ^6^ Key Laboratory of Carcinogenesis and Translational Research (Ministry of Education), Department of Pathology, Peking University Cancer Hospital and Institute, Beijing, China

**Keywords:** single-cell RNA sequencing, molecular, cellular, progression, lung adenocarcinoma

## Abstract

Pure ground glass nodules (GGNs) and solid nodules (SNs) represent early and relatively late stages of lung adenocarcinoma (LUAD) in radiology, respectively. The cellular and molecular characteristics of pure GGNs and SNs have not been comprehensively elucidated. Additionally, the mechanism driving the progression of lung adenocarcinoma from pure GGN to SN in radiology is also elusive. In this study, by analyzing the single-cell transcriptomic profiles of 76,762 cells from four pure GGNs, four SNs, and four normal tissues, we found that anti-tumor immunity mediated by NK and CD8+T cells gradually weakened with the progression of LUAD and humoral immunity mediated by plasma B cells was more active in SNs. Additionally, the proliferation ability of some special epithelial cell increased during the progression process from pure GGN to SN. Furthermore, stromal cells and M2 macrophages could assist the progression of LUAD. Through comprehensive analyses, we revealed dynamic changes in cellular components and intercellular interactions during the progression of LUAD. These findings could facilitate our understanding of LUAD and discovery of novel therapeutic targets.

## Introduction

Lung cancer is the leading cause of cancer-related mortality, among which lung adenocarcinoma (LUAD) represents the major histological subtype, both globally and in China (Chen et al., 2016; Siegel et al., 2021). The disease spectrum has gradually shifted with population-based lung cancer screening. More and more early-stage LUADs manifesting as ground glass nodule (GGN) in radiology have been detected (Gao et al., 2017), and they are further classified into pure GGNs and mixed GGNs, which represent a special entity characterized by slow growth and indolent non-invasive features (Chang et al., 2013). Previous studies have demonstrated that pure GGNs could develop into mixed GGNs and then solid nodules (SNs) over time (Silva et al., 2012; Cho et al., 2016). Patients with GGN can harbor excellent survival by surgical resection, and some GGNs do not affect the lifespan of patients under regular surveillance. However, LUADs manifesting as SN are often more aggressive and exhibit a higher recurrence rate after resection (Zhang et al., 2020). Additionally, although multiple treatment modalities, including surgical resection, chemotherapy, targeted therapy, and immunotherapy, have significantly improved the clinical outcome of patients with LUAD, treatment effect varies considerably ranging from permanent cure to rapid progression (Hirsch et al., 2017; Billan et al., 2020). That is predominantly attributed to the limited understanding of the underlying mechanisms of tumor progression and inter-tumoral and intra-tumoral cellular and molecular heterogeneity of LUAD (Hua et al., 2020; Yuan et al., 2021).

Conventional bulk sequencing has uncovered abundant molecular aberrations that drive carcinogenesis and the progression of LUAD. For instance, Chen et al. revealed that the frequency of TP53, arm-level copy number alterations, and HLA loss of heterozygosity increased during the progression from pre-invasive lesion to invasive LUAD (Chen et al., 2019). However, LUAD is composed of various types of cells, including epithelial cells, stromal cells, and immune cells, and they constitute a complex ecosystem (Lambrechts et al., 2018). Due to methodological limitations of bulk sequencing, important molecular alterations in cancer cells may be undervalued. Additionally, rare but functionally important cell subpopulations (e.g., cancer stem cells and tumor-infiltrated immune cells) may not be identified. In order to comprehensively decipher the underlying molecular mechanism and reveal the heterogeneity of LUAD, particularly at different development stages, more advanced techniques, such as single-cell RNA sequencing (scRNA-seq), are required.

Recent advances in scRNA-seq enable exploring the microenvironment and intra-tumoral heterogeneity of human solid tumors and allow for assessment of heterogeneous cell populations at single-cell resolution (Lei et al., 2021). Notably, scRNA-seq has also been employed to dissect the molecular characteristics of lung cancer, including LUAD (Lavin et al., 2017; Guo et al., 2018). A series of pioneering studies comprehensively mapped the immune landscape of LUAD, and revealed unprecedented intra-tumoral heterogeneity. This provided deeper insights into the biology of LUAD and guided immunotherapy design (Lavin et al., 2017). One recent study of advanced metastatic LUAD revealed the molecular and cellular reprogramming during metastasis, which provided diagnostic and therapeutic targets (Kim et al., 2020). Another study provided single-cell transcriptomic profiling of subsolid nodules and facilitated the understanding of their indolent nature (Xing et al., 2021). These studies have decoded comprehensive cellular and molecular characteristics of special stages of LUAD or individual cell types, such as T cells. Nevertheless, it is still ambiguous about the dynamic evolutionary procedure of LUAD from pure GGNs to SNs, especially at single-cell resolution. A comprehensive understanding of this procedure is of significance to develop reasonable and effective treatment strategies for patients at different stages of LUAD.

In the present study, we performed scRNA-seq on fresh surgical specimens from eight resectable LUADs, among which four were presented as pure GGNs and the other four were SNs on computed tomography (CT) image. They represent different development phases of LUAD in radiology. By comprehensively analyzing their cellular and molecular profiles, we revealed dynamic changes in cellular components and intercellular interactions during the evolution of LUAD. We found that anti-tumor immunity mediated by NK and CD8^+^ T cells gradually weakened and humoral immunity mediated by plasma B cells was more active in late stages. These findings may facilitate our understanding of LUAD and the discovery of novel therapeutic targets.

## Materials and methods

### Patients and specimens

Eight treatment-naïve patients with early-stage LUAD in radiology who underwent radical resection between December 2018 and November 2019 at the Department of Thoracic Surgery II, Peking University Cancer Hospital & Institute were enrolled in this study. This study was conducted in accordance with the Declaration of Helsinki (as revised in 2013) and approved by the Ethics Committee of Peking University Cancer Hospital and Institute (Institutional Review Board No. 2019KT59). All patients provided written informed consent before surgery. Demographic and clinicopathological characteristics of these patients are listed in Supplementary Table S1.

Tumor tissues were collected from four patients with pure GGN (WDF, WST, JYS, and YL) and four patients with SN (HYP, LHY, QDL, and CGC). Additionally, non-cancerous adjacent normal tissues were collected from one patient with SN (LHY), one with pure GGN (YL) and two other patients (YLF and LY). Fresh tumor or non-cancerous adjacent normal tissues were collected immediately after a qualified pathologist confirmed the pathological diagnosis of these nodules *via* fast frozen pathology during surgery. Non-cancerous adjacent normal tissues were usually collected at the edge of the resected lobe and were macroscopically normal. In the cases of sublobar resection, they were collected > 2 cm apart from the tumor edge.

To validate the results of scRNA-seq, formalin-fixed paraffin-embedded (FFPE) tumor tissues from another cohort comprising fifteen patients with pure GGN and fifteen with SN who also underwent surgery at the Department of Thoracic Surgery II, Peking University Cancer Hospital & Institute were evaluated for the expression of selected markers at protein level using immunohistochemistry (IHC), and their brief clinicopathological characteristics of them are listed in Supplementary Table S2 (validation cohort). All patients provided written informed consent before surgery.

### Tissue dissociation and sequencing

Fresh tissues were preserved in tissue storage solution (Miltenyi Biotech, Cat. No. 130-100-008) and were transported on ice to preserve viability, and all tissues were dissociated within 2 h after surgery. After being washed 3 times with phosphate buffered saline (PBS; Gibco, Cat. No. 8120002), tissues were cut into small pieces of 1–2 mm^3^ in size and then transferred to a gentleMACS C tube containing 5 ml enzyme mix (Miltenyi Biotech, Cat. No. 130-095-929). The gentlMACS programs h_tumor_01, h_tumor_02, and h_tumor_02 were run with two 30-min incubations on MACSmix™ Tube Rotator at 37 °C. Then, the samples were centrifugated, and the resuspended samples were filtered to a 50 ml tube through MACS SmartStrainer (70 μm). The filtered suspension was centrifuged at 300 g for 7 min, and the supernatant was completely aspirated. After that, red blood cells were removed by RBC lysis buffer (BD, Cat. No. 349202), and cell suspension was centrifuged at 300 g for 7 min again. The cell pellet was used for the next experiments. Cell suspension was processed to prepare scRNA-seq libraries by Chromium Single cell 3′ Reagent V2 kits with a cell recovery target of 8,000, following the manufacturer’s instructions (10× Genomics, Pleasanton, CA, United States). Libraries were sequenced on an Illumina Xten.

### Raw data processing and quality control

Raw sequencing data were mapped to the GRCh38 human reference genome and quantified at gene level to produce gene expression matrix cross-cells with Cell Ranger toolkit (v3.1.0). The expression matrixes were first converted to Seurat object with Seurat (v4.0.3). Next, cells with less than 200 genes or more than 10% mitochondrial-derived unique molecular identifiers (UMI) were considered as low-quality cells and filtered out. Additionally, those with more than 5,000 gene expressions were also excluded, which were thought to be doublets.

### Unsupervised dimensional reduction and clustering

The gene expression matrix was normalized by the NormalizedData function. The cell cycle score was obtained by CellCycleScoring function and then the difference between G2M and S-phase score was eliminated. Next, ElbowPlot function was employed to identify the true dimensionality, and the top 30 principal components (PCs) were involved in RunPCA function. Then, these cells were clustered by FindNeighbors and Findclusters function with resolution set as default. Finally, cell clusters were displayed as Uniform Manifold Approximation and Projection (UMAP) plot with Dimplot function. Findallmarkers function was used to find the highly expressed or differentially expressed genes. According to the canonical marker genes of each cluster, these cell clusters were annotated.

### Estimation of CNV

The R package “inferCNV” (v1.8.1) was used to infer the CNV of each cell type, and the cells derived from stromal cells and immune cells were used as normal controls. The R code was provided in https://github.com/broadinstitute/inferCNV with the default parameters except for cut-off = 0.1.

### Inference of cell state through trajectory analysis

The Monocle2 (v2.20.0) package was used to analyze single-cell trajectories to discover the cell-state transitions. The Seurat object was first converted to the monocle object, and then the significantly changed genes calculated by differentialGeneTest function were used to determine the differential cell states. The state where most normal cells clustered was considered as the root state and used to specify the start of the pseudotime trajectory. The position of cells on the trajectory was determined by orderCells function. The visualization of cell differentiation and gene expression changes with pseudotime was performed by function plot_cell_trajectory, plot_genes_in_pseudotime and plot_pseudotime_heatmap, respectively.

### Cell–cell interaction network

The interaction network between cells was analyzed by using CellPoneDB2 (https://www.cellphonedb.org/). The gene expression matrix of single cells and the corresponding cell type information were used as input files to infer the potential interaction strength between every cell type, with interaction set as default (interactions = 1,000). The interactions between cells were evaluated by means and *p*-value from the output file (*p* ≤ 0.05).

### Regulons network analysis

SCENIC (v1.2.4) was used to analyze the regulons networks in our data based on single-cell RNA datasets and produce the expressed regulons of each cell. Next, upregulated or downregulated regulons of different clusters were calculated by limma package (v3.48.3) with adj. *p*-value < 0.05 and then were used for subsequent analysis.

### Public data analysis

RNA-seq data and clinical information of LUAD from the Cancer Genome Atlas (TCGA) database were downloaded from UCSC Xena (https://xena.ucsc.edu/). The average expression differences between tumor and normal tissues in TCGA of special maker genes identified from our samples were compared by *t*-test. Additionally, the non-negative matrix factorization (NMF) model was used to reduce dimensionality of expression matrix of marker genes among TCGA LUAD cohort, and survival curve was constructed by the R package “Survminer” (0.4.9) and “Survival” (3.2–13) to assess the relationship between special marker genes expression and prognosis. The average expression differences between two gene expression matrixes generated by NMF was compared by *t*-test.

### Pathway analysis

The marker genes of each cell subsets and the differentially expressed genes of tissues of different origin were, respectively, calculated through FindAllmarkers function (*p* ≤ 0.05, logFC ≥ 0.25) and limma package. The functional enrichment was performed on GO enrichment. Gene set variation analysis (GSVA) was used to assess differential pathways, and the activity scores of special cells were also calculated by GSVA (v1.40.1).

### Inferring malignant cells

The CNV expression matrix of epithelial cells of each sample was obtained by R package inferCNV. The CNV score of each cell was calculated by the mean square of CNV obtained by “inferCNV.” The cells were sorted according to the CNV score, and the top 5% cells were regarded as the reference cells. The average CNV value of top 5% cells was calculated firstly, and then the correlation coefficient of CNV scores between each cell and average CNV was calculated as described above. The cells with the correlation coefficient over than 0.3 were regarded as malignant cells.

### Immunohistochemistry

Serial 4 μm sections of FFPE samples were cut onto glass slides and subjected to IHC staining following standard protocol. Firstly, the slides were deparaffinized by using xylenes and ethanol gradient. Antigen retrieval was performed in citric acid buffer in a 95°C water bath for 20 min, and then the slides were incubated in 3% H_2_O_2_ for 25 min, then added with 3% BSA for 30 min at room temperature to block the non-specific binding sites. After that, the slides were incubated with primary antibodies followed by HRP-linked secondary antibodies and diaminobenzidine (DAB, Servicebio, Cat. No. G1211) staining. After counterstaining with hematoxylin, slides were dehydrated with sequential ethanol washes.

The following antibodies were used to detect the respective proteins: anti-RGS5 (rabbit, 1:500, ab196799, ABCAM), anti-Ki67 (mouse, 1:500, #9449, CST), anti-HLA-DR (rabbit, 1:500, ab92511, ABCAM), anti-TOP2A (mouse, 1:500, #12286, CST), anti-PTGDS (rabbit, 1:200, PA018969HA01HU, Cusabio Technology), and anti-Syndecan-1 (CD138, rabbit, 1:500, ab128936, ABCAM).

The intensity of RGS5, TOP2A, PTGDS, and Sydecan-1 was scored as 0 (no staining), 1 (weak), 2 (moderate), and 3 (strong) by experienced pathologists. The Ki-67 expression level was estimated by counting the ratio of positive tumor cells in representative regions. As for HLA-DR, the slides were scanned by whole slide digital scanning using a digital pathology scanner (Aperio VERSA, Leica Biosystems, Buffalo Grove, IL, United States), and scoring was assessed on an Aperio Scanscope (Aperio Technologies, United States). The average positive cell ratio was measured in 8 intra-tumoral non-overlapping fields using fixed areas of 0.078 square millimeters.

## Results

### Single-cell transcriptomic profiling of pure ground glass nodules and solid nodules

**FIGURE 1:**
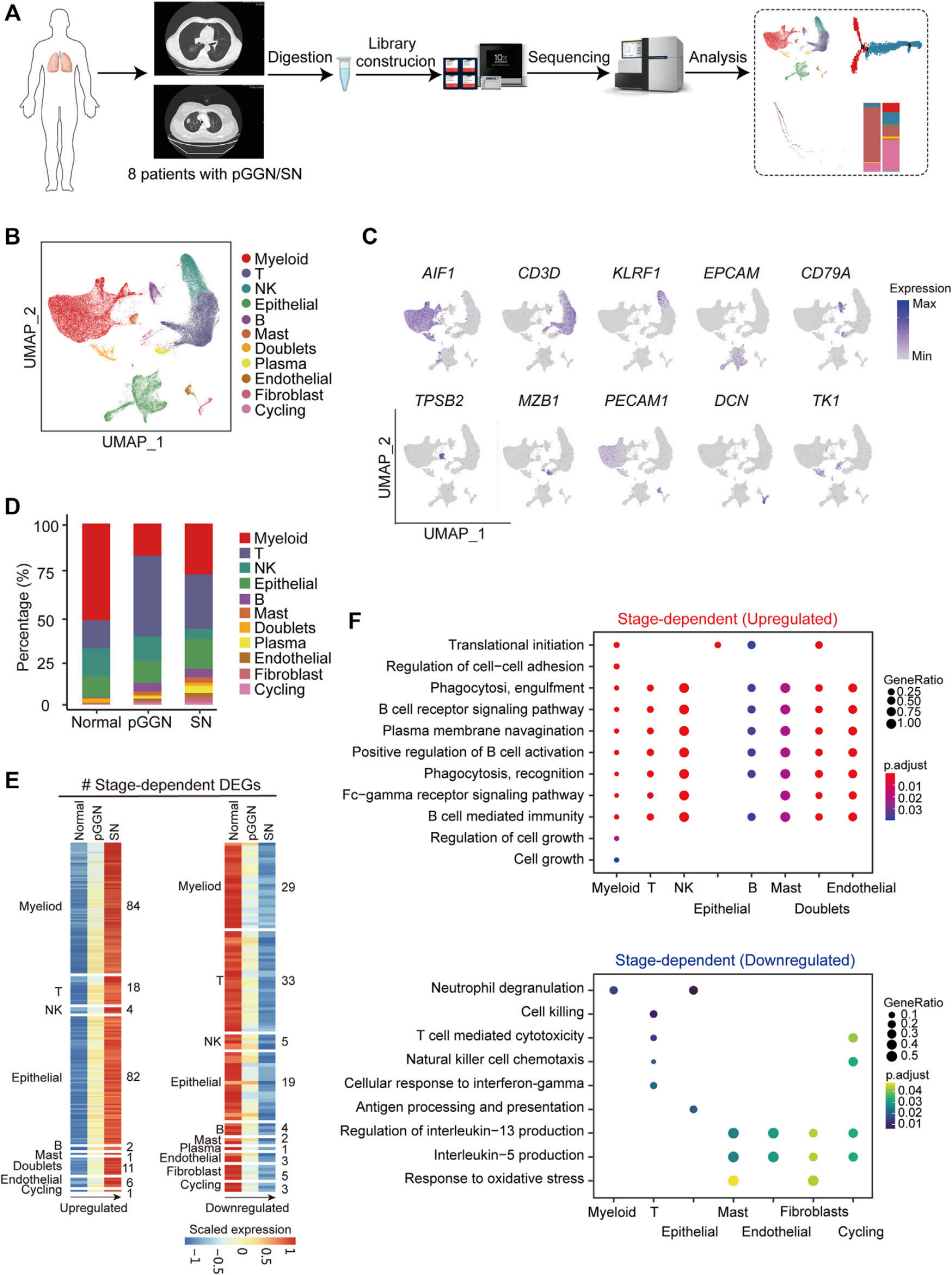
Single-cell transcriptomic profiling of pure GGN and SN. **(A)** Schematic diagram of experimental flow. Single-cell RNA sequencing was applied to cells derived from 4 pure ground glass nodules (GGNs), 4 solid nodules (SNs) and 4 normal tissues. **(B)** Uniform manifold approximation and projection (UMAP) plot of 76,762 cells from 8 tumors and 4 adjacent normal tissues, colored by 11 major cell types. **(C)** UMAP plot of canonical cell type markers, colored by gene expression. **(D)** The proportion of major cell types among different tissue origins, colored by the major cell types as shown in **(B)**. **(E)** The expression of stage-dependent upregulated (left) and downregulated (right) genes of each cell type. **(F)** Enriched GO terms (*p* < 0.05) of upregulated genes (upper) and downregulated gene (lower) of each cell type. The color indicates the adjusted *p-*value, and the size of dot indicates gene ratio.

To elucidate the cellular and molecular characteristics of different developmental phases of LUAD, tumor tissues from four pure GGNs and four SNs were prospectively collected for scRNA-seq in this study. Additionally, non-cancerous adjacent normal tissues from one patient with pure GGN, one with SN, and two other patients were also obtained as controls (Supplementary Table S1). The CT images of each tumor are shown in Supplementary Figure S1A. Single-cell solutions and DNA libraries of each tissue were carefully constructed using 10X Genomics and were then sequenced. The single-cell transcriptomic profiles of normal tissues, pure GGNs, and SNs were characterized and compared using comprehensive bioinformatic analyses (Figure 1A).

A total of 76,762 high-quality cells were obtained, including 22,053 cells from pure GGNs, 21,306 cells from SNs, and 33,403 cells from normal tissues. The correlation coefficient between samples calculated in the same group was high (≥0.89), suggesting that the batch effect among samples was relatively small (Supplementary Figure S1). According to the expression of canonical marker genes, all of these cells were assigned to 10 well-known cell types (Figure 1B): myeloid cells (*LYZ*+, *CD163*+, and *CD68* ^+^), T cells (*CD3D*+ and *TRAC*+), NK cells (*NKG7*+, *GNLY*+, and *KLRF1*+), epithelial cells (*EPCAM*+, *KRT19*+, and *KRT18*+), B cells (*CD79A*+), mast cells (*MS4A2*+, *TPSB2*+, and *GATA2*+), plasma B cells (*MZB1* and *IGHA1*+), endothelial cells (*PECAM1*+ and *FLT1*+), fibroblasts (*COL1A2*+, *COL6A2*+, and *DCN*+), and cycling cells (*TK1+, MKI67+*, and *TOP2A+*) (Figure 1C; Supplementary Figure S1C). It was observed that the proportion of plasma B cells, B cells, fibroblasts, and endothelial cells increased sequentially from normal tissues to pure GGNs and then to SNs, while that of NK cells decreased (Figure 1D). The proportion of T cells was relatively higher and that of myeloid cells was lower in pure GGNs than in SNs. When we classified immune cells into innate immunity and adaptive immunity, the adaptive immune response was significantly activated, whereas the innate immunity was significantly inactivated in both pure GGNs and SNs compared with that of normal tissues (Supplementary Figure S1D), indicating that innate immune response mainly mediated by NK cells might gradually weaken during the progression of LUAD. However, adaptive immune response was more highly activated in pure GGNs than in SNs, suggesting that adaptive immune response to tumors was relatively stronger in the earlier stage of LUAD development (Supplementary Figure S1D).

Progression of LUAD was accompanied by transcriptome changes. In this study, a total of 1,113 upregulated genes and 1,240 downregulated genes (|avg_logFC| ≥ 0.25 and *p*_val_adj ≤0.05) were identified during the progression from pure GGNs to SNs (Supplementary Figure S1E). We compared differentially expressed genes (DEGs) among the total cells from three types of tissues and obtained 34 upregulated and 29 downregulated genes related to the progression of LUAD (Supplementary Figure S1F). Furthermore, we comprehensively characterized DEGs according to distinct cell types with the progression of LUAD, namely, stage-dependent DEGs, and identified 209 upregulated DEGs in 9 cell types and 104 downregulated in 10 cell types (Figure 1E). Gene ontology (GO) enrichment analysis revealed that the upregulated stage-dependent DEGs were mainly related to cell growth, regulation of cell–cell adhesion, B cell receptor signaling pathway, phagocytosis and other pathways, while the downregulated stage-dependent DEGs were mainly related to neutrophil degranulation, cell killing response, T cell-mediated cytotoxicity, and response to oxidative stress (Figure 1F).

### Proliferation ability of special epithelial cells increases during the progression of lung adenocarcinoma

**FIGURE 2:**
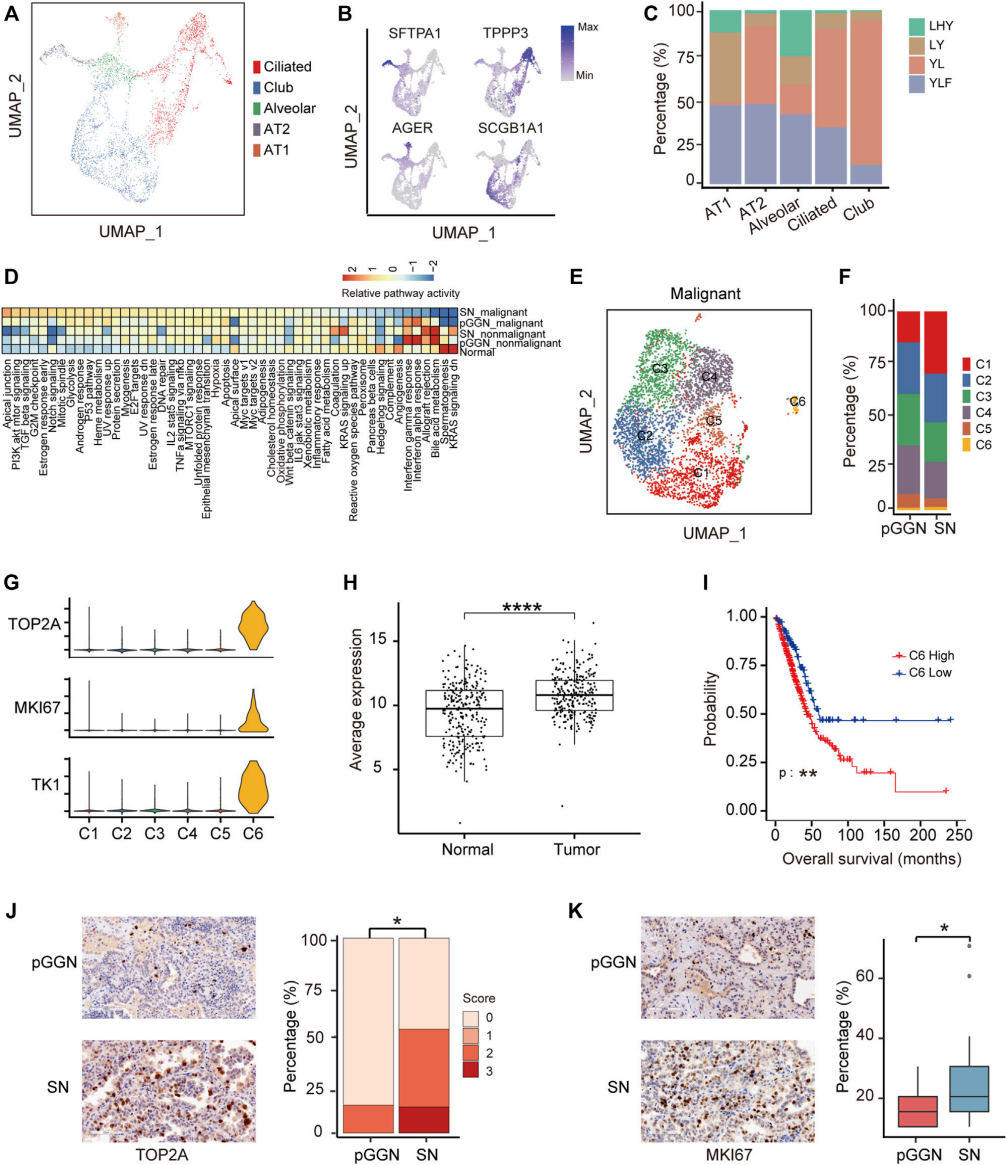
Proliferation ability of special epithelial cells increases during the progression of LUAD. **(A)** UMAP plot of epithelial cells from 4 normal adjacent tissues, colored by 5 cell subtypes. **(B)** UMAP plot of canonical cell type markers, colored by gene expression. Four highly expressed genes were used to define 5 cell types, of which one cell type is a mixture. **(C)** The proportion of normal samples among each cell type, colored by patient origins. **(D)** The enrichment of hallmark pathways by GSVA analysis among normal epithelial cells, nonmalignant cells, and malignant cells. The scores were sorted by relative pathway activity. **(E)** UMAP plot of epithelial malignant cells, colored by 6 cell subsets. Epithelial cells from tumor tissues were divided into malignant cells and nonmalignant cells by inferCNV. **(F)** The proportion of cell types among each tissue origin, colored by cell subtypes. **(G)** The expression of proliferative markers (*TOP2A*, *MKI67*, and *TKI*) in epithelial malignant cells subsets. **(H)** The average expression of marker genes of C6 in TCGA LUAD cohort. It was significantly higher in tumor tissue than in normal tissue. **(I)** The survival curves of two groups of TCGA LUAD patients with high and low expression of C6 cluster marker genes. The *p*-value was calculated by two-sided log-rank test. **(J)** The expression of TOP2A in pure GGNs and SNs by immunohistochemical examination, and the left was the representative images. Score 0, 1, 2, and 3 represents no, weak, moderate, and strong staining, indicating the relative expression level of TOP2A from low to high. TOP2A is highly expressed in SNs than in pure GGNs. **(K)** The expression of MKI67 in pure GGNs and SNs by immunohistochemical examination, and the left was the representative images. The ratio of positive expressed tumor cells in pure GGNs and SNs was compared. The comparison in **(H,J)** and **(K)** was performed using *t*-test. *, *p* ≤ 0.05; **, *p* ≤ 0.001; ***, *p* ≤ 0.0001. A *p*-value < 0.05 was considered statistically significant.

A total of 3,969 epithelial cells from normal tissues were obtained, and based on canonical marker genes, these epithelial cells were subclustered into 5 subsets, including ciliated cells (*TPPP3*+), club cells (*SGCB1A1*+), alveolar cells (*SFTPA1*+ and *AGER*+), alveolar type II cells (AT2; *SFTPA1*+), and alveolar type I cells (AT1; *AGER*+) (Figures 2A,B). Among them, alveolar cells were the mixture of AT1 and AT2, expressing SFTPA1 and AGER simultaneously. The distribution of these cells in each sample is shown in Figure 2C. The 6,215 epithelial cells from tumor tissues were divided into malignant and nonmalignant cells based on the CNV expression matrix by inferring CNV (Supplementary Figure S2A). Obviously, the proportion of malignant cells in SNs was higher than that in pure GGNs (Supplementary Figure S2B,C). Totally, 756 genes were upregulated and 487 genes were downregulated in malignant cells compared with nonmalignant cells (|avg_logFC| ≥ 0.25 and *p*_val_adj ≤0.05) (Supplementary Figure S2D). GO enrichment analysis showed that the response to hypoxia, epithelial tube morphogenesis, and regulation of protein serine/threonine kinase activity pathways were upregulated in malignant cells. Notably, the regulation of humoral immune response, neutrophil-mediated immunity, and regulation of T-cell activation pathways were activated in nonmalignant cells (Supplementary Figure S2E; Supplementary Table S3). Furthermore, gene set variation analysis (GSVA) inferred that the function of the malignant cells from tumors, was different from that of epithelial cells from normal tissues and nonmalignant cells from tumors (Figure 2D). The G2M checkpoint, TGF beta signaling, and glycolysis were upregulated in malignant cells from SNs. Spermatogenesis and KRAS signaling pathways were upregulated in epithelial cells from normal and nonmalignant cells from tumors, suggesting that the proliferation ability of malignant cells from SNs was enhanced.

To further assess malignant cells, they were re-clustered into six cell subsets (Figure 2E). Among them, the proportion of C1 and C6 increased from pure GGNs to SNs, while those of C2, C3, C4, and C5 decreased (Figure 2F). The marker genes of C1–C5 were involved in similar functions, such as positive regulation of cell–cell adhesion and T-cell activation, while marker genes of C6 were mainly related to the cell cycle G2/M phase transition and positive regulation of the cell cycle (Supplementary Figure S2F). The expression of marker genes of C6 cluster cells, including a total of 276 genes such as *TOP2A*, *MKI67*, and *TK1* (Figure 2G), was higher in tumor tissues than in normal tissues from TCGA database (Figure 2H). In addition, patients with LUAD from TCGA with higher expression of these genes in tumor tissues had a shorter overall survival compared with those patients with lower expression (Figure 2I; Supplementary Figure S2G), suggesting that these genes could serve as promising prognostic biomarkers. Furthermore, the expression of TOP2A and MKI67 was also higher in the SN group than in the pure GGN group at protein level as estimated by immunohistochemistry (IHC) in the validation cohort (Figures 2J,K).

Additionally, we extracted epithelial cells from tumor tissues to infer the evolutionary trajectory and found that nonmalignant cells were located in the early pseudo time (Supplementary Figure S2H). The heatmap of gene changes accompanied by pseudo time showed that *TPPP3*, *SCGB3A1*, and MHC family genes such as *HLA−DRB5* and *HLA−DRB1* were upregulated in the early pseudo time, and that *SOX4* and *SFTPA1* were upregulated in the end of the pseudo time (Supplementary Figure S2I). These indicated that these genes might be involved in the malignant transformation of epithelial cells.

### Stromal cells assist the progression of lung adenocarcinoma

**FIGURE 3:**
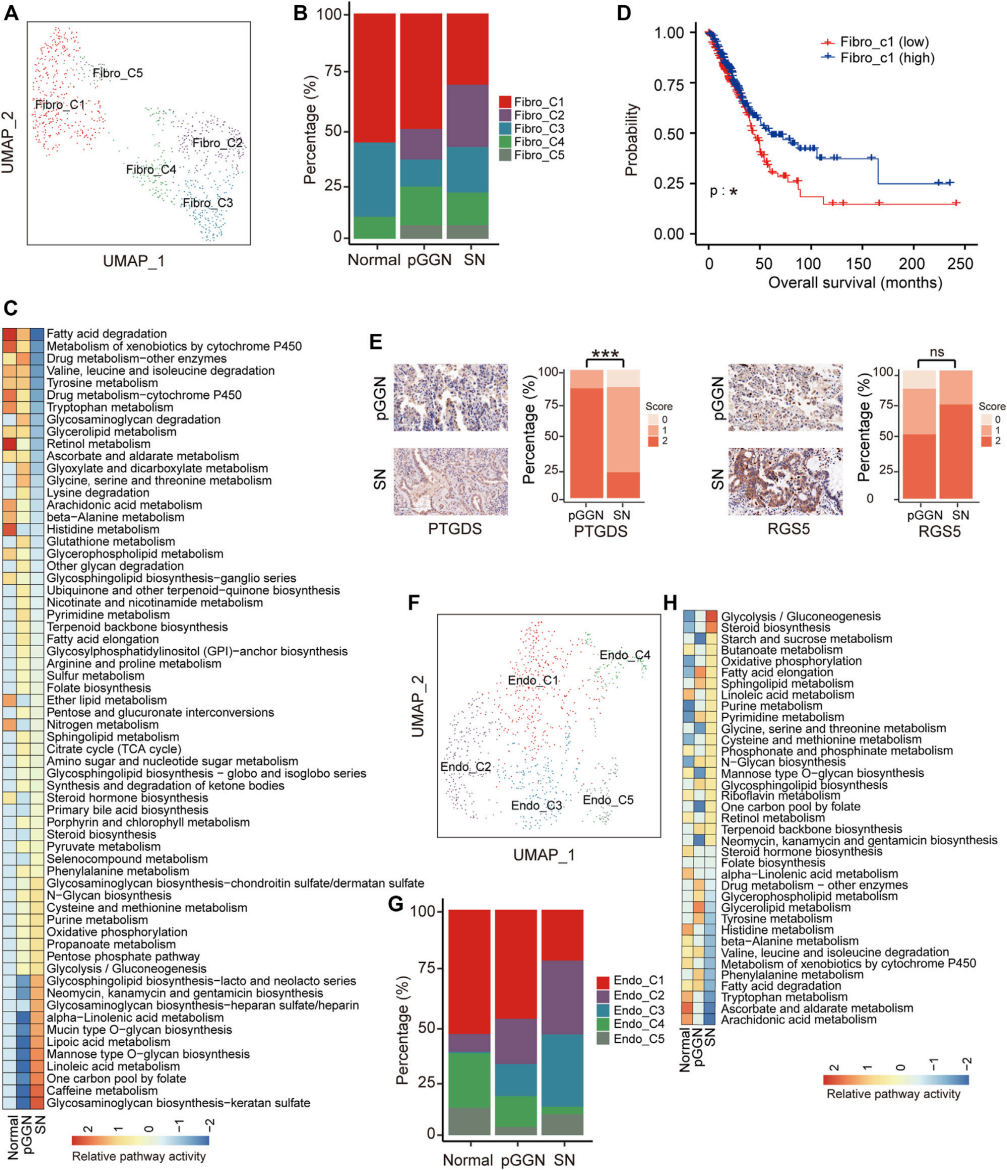
Stromal cells assist progression of tumor during the progression of LUAD. **(A)** UMAP plot of fibroblasts, colored by 5 cell subtypes. **(B)** The proportion of major cell subsets among each tissue origin. **(C)** Heatmap showing the activity of metabolic pathways for fibroblasts, among pure GGNs, SNs, and normal tissues. Many metabolic pathways were upregulated in fibroblasts derived from SNs. **(D)** The survival curves of two groups of TCGA LUAD patients with high and low expression of Fibro-C1 cluster marker genes. The *p*-value was calculated by two-sided log-rank test. **(E)** The expression of PTGDS and RGS5 in the pure GGNs and SN tissues by immunohistochemical examination. The comparison was performed using *t*-test. **(F)** UMAP plot of endothelial cells, colored by 5 major cell types. **(G)** The proportion of major cell subsets among each tissue origin. **(H)** The enriched metabolic pathways by GSVA analysis in endothelial cells from different tissue origin. The scores were sorted by pathway activity in malignant cells from SN. ns, *p* > 0.05; *, *p* ≤ 0.05; **, *p* ≤ 0.001; ***, *p* ≤ 0.0001. A *p*-value < 0.05 was considered statistically significant.

A total of 923 fibroblasts were divided into five subsets (Figure 3A; Supplementary Figure S3A), including inflammatory cancer-associated fibroblasts (Fibro-C1, also known as iCAFs; *PTGDS*+) secreting cytokines such as *CXCL12*, *CCL2*, and *CXCL14* (Supplementary Figure S3B **)**, pericytes (Fibro-C2; *RGS5*+ and *PDGFRB*+), myofibroblasts (Fibro-C3; *ACTA2*+), antigen-presenting CAFs (Fibro-C4; *CXCR4*+ and *HLA*−*DRA*+), and other fibroblasts (Fibro-C5; *COL1A1*+ and *MMP2*+). The abundance of pericytes (Fibro-C2) increased sequentially from normal tissues to pure GGNs, and then to SNs, while that of iCAFs (Fibro-C1) decreased (Figure 3B). The proportion of Fibro-C4 was the highest in pure GGNs, and that of Fibro-C3 was the lowest in pure GGNs. Notably, Fibro-C5 existed only in tumor tissues (Figure 3B). GSVA analysis revealed that metabolic pathways, such as glycosaminoglycan biosynthesis and glycolysis were upregulated in fibroblasts derived from SNs (Figure 3C).

Previous studies have demonstrated that iCAFs promote anti-tumor immunity (Biffi and Tuveson, 20212019). Hence, the decrease of iCAFs (Fibro-C1) implied that the anti-tumor immune response might gradually decrease with tumor progression. (Figure 3B). Additionally, higher expression of marker genes of iCAFs was correlated with longer overall survival in the TCGA LUAD cohort (Supplementary Figure S3C; Figure 3D). Previous studies have reported that pericytes (Fibro-C2) significantly contribute to cancer invasion and metastasis by transitioning to fibroblasts (Hosaka et al., 2016; Zeltz et al., 2020). The proportion of Fibro-C2 increased sequentially from normal tissues to pure GGNs, and then to SNs, suggesting that the ability of cancer invasion and metastasis mediated by Fibro-C2 gradually increased with tumor progression, and targeting Fibro-C2 might offer a new treatment option to inhibit cancer metastasis. We detected the expression of *PTGDS* and *RGS5*, the marker genes of Fibro-C1 and Fibro-C2, respectively, by IHC in the validation cohort, and found that PTGDS was highly expressed in pure GGNs and the expression of RGS5 was lower in pure GGNs than that in SNs (Figure 3E), which was consistent with the results of scRNA-seq. Fibro-C4 expressed high levels of MHC family genes, such as *HLA-DRA*, indicating the features of antigen presentation (Supplementary Figure S3A). The high proportion of Fibro-C4 in pure GGNs indicated that the antigen presentation process was more active in the initial stage of LUAD.

Except for fibroblasts, a total of 937 endothelial cells were also obtained, which were subclustered into five subsets (Figure 3F; Supplementary Figure S3D), including extra-alveolar capillary ECs (Endo-C1; *EDN1*+), tumor ECs (Endo-C3; *INSR*+), and other ECs (Endo-C2, *ICAM1+*; Endo-C4, *MAP1B*+; Endo-C5, *CXCR4*+). Specifically, the proportion of Endo-C1 and Endo-C4 decreased gradually, whereas that of Endo-C2 increased gradually with tumor progression. Endo-C5 was the least abundant cells in pure GGNs (Figure 3G). GO analysis showed that Endo-C1 exhibited the characteristics of endothelium development, and pathways such as extracellular structure organization were upregulated in Endo-C4. Notably, negative regulation of the immune system process and regulation of vasculature development were upregulated in Endo-C2 and Endo-C3, respectively (Supplementary Figure S3E). In addition, a comparison of the metabolic pathways by GSVA analysis between endothelium from normal tissues, pure GGNs and SNs revealed that signaling pathways that could assist tumor growth, such as glycolysis, were enriched in the endothelium in SNs (Figure 3H).

### Progression of lung adenocarcinoma is promoted by M2 macrophages

**FIGURE 4:**
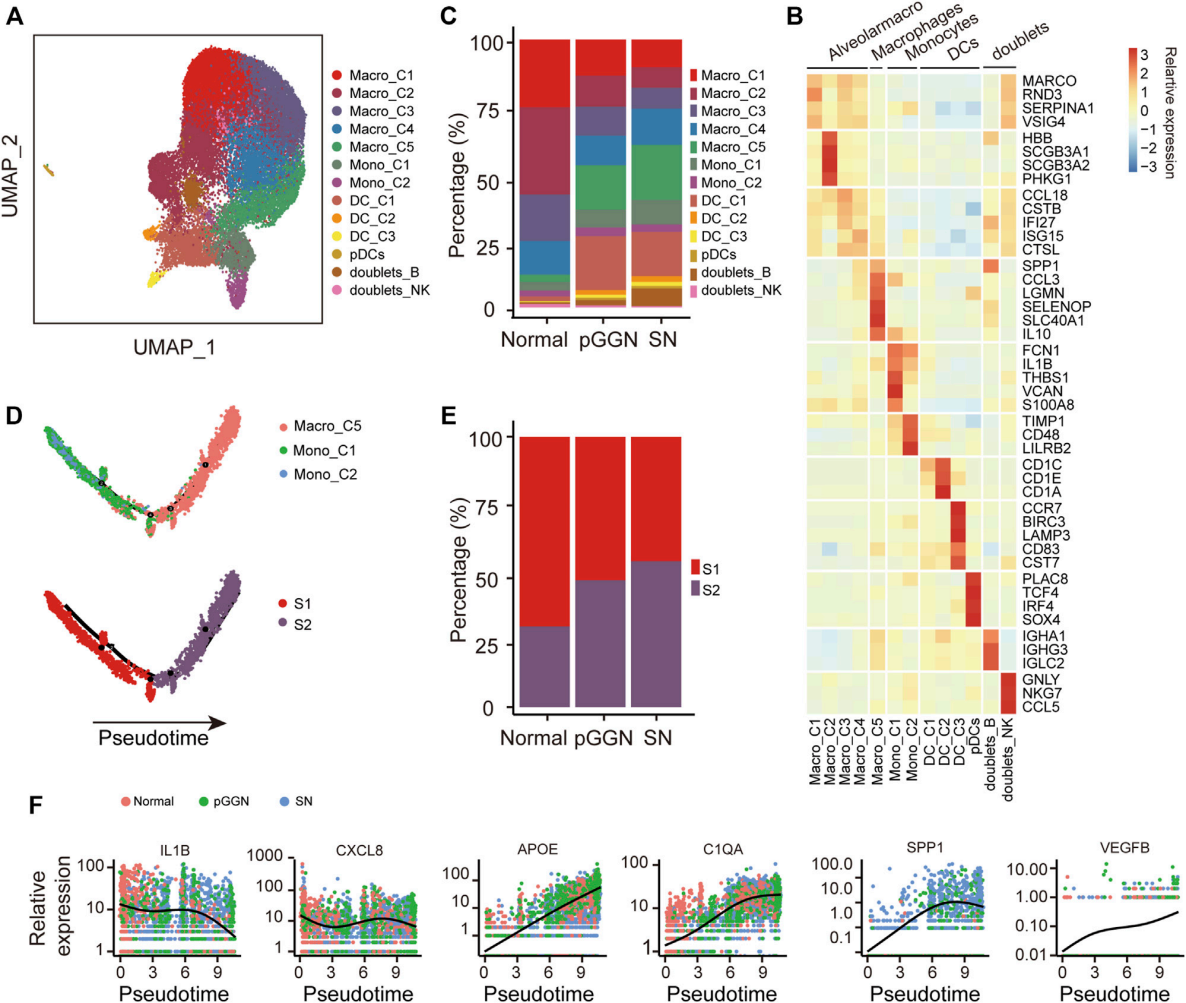
Progression of LUAD promoted by M2 macrophages. **(A)** UMAP plot of myeloid cells, colored by 14 cell subtypes. **(B)** Heatmap showed the expression of canonical marker genes in each myeloid cell subset. **(C)** The proportion of cell subsets among each tissue origin, colored by cell types. **(D)** Unsupervised transcriptional trajectory of monocytes and macrophages from Monocle2, colored by cell states (lower) and cell types (upper). **(E)** The proportion of cell states of the trajectory among each tissue origin. Red and purple represent S1 and S2 cell state, respectively. **(F)** The expression changes of M1-related genes (i.e., *IL1B* and *CXCL8*), M2-related genes (i.e., *APOE* and *C1QA*), tumor malignancy-related genes (i.e., *SPP1*), and angiogenesis-related genes (i.e., *VEGFB*) with pseudo time trajectory. The relative expression was normalized by raw counts. The smooth black line represents the kinetic patterns along the pseudo time trajectory, which is modeled by Monocle2. Pure GGNs, SNs, and normal tissues are labeled using different colors.

Myeloid cells play a critical role in maintaining tissue homeostasis and regulating inflammation in the lung (Bissonnette et al., 2020; Hou et al., 2021). Additionally, previous studies also confirmed that they had impacts on the progression of LUAD (Lavin et al., 2017; Zheng et al., 2020). A total of 27,758 myeloid cells were detected in this study (Figures 4A,B), and they were subclustered into 13 subsets based on canonical marker genes, including alveolar macrophages (AM; *MARCO*+; including 4 subsets), macrophages (Macro-C5; *LGMN+* and *IL10+*), monocytes (*FCN1*+ and *IL1B*+; including 2 subsets), dendritic cells (DC; *CD1C*+; including 3 subsets), pDCs (*TCF4*+), and doublets (including 2 subsets). Among them, the doublets-B subset expressed both B cell markers and myeloid markers and the doublets-NK subset expressed both myeloid markers and NK cell markers. Notably, we found that AMs were mainly derived from normal tissues, whereas the abundance of Macro-C5 increased from normal tissues to pure GGNs, and then to SNs (Figure 4C).

Numerous studies have demonstrated that macrophages are involved in tumorigenesis and drug resistance (Schmall et al., 2015; Wang et al., 2017). Monocytes and macrophages are both the members of the mononuclear phagocyte system, and monocytes are the precursors of macrophages (Ginhoux and Jung, 2014). In order to understand the relationship between monocytes and macrophages in the progression of LUAD, an unsupervised trajectory analysis was performed through the Monocle2 package to assess transcriptional changes from monocytes to macrophages. We found that this trajectory evolved from monocytes to macrophages, and it consisted of two transcriptional states (S1 and S2, Figure 4D). S1 was observed to be distinctly positioned at the first half of the trajectory, while S2 was located at the second half of the trajectory. Intriguingly, the cells of S1 gradually decreased from normal tissues to pure GGNs, and then to SNs, whereas those of S2 gradually increased and were significantly enriched in SNs (Figure 4E). These results suggest that monocytes differentiated into macrophages along with the progression of LUAD. Additionally, along with the trajectory, the expression of M1 macrophage maker genes, including *IL1B* and *CXCL8*, decreased, while the expression of M2 macrophage marker genes, including *APOE* and *C1QA*, increased (Figure 4F), indicating that macrophages might gradually polarize from M1 to M2 as the trajectory evolved. Previous studies have demonstrated that M2 macrophages are involved in wound repair, tissue fibrosis, and angiogenesis. In this study, the expression of *VEGFB* also gradually increased as the trajectory evolved (Figure 4F), and the expression of *SPP1*, which could promote tumor progression (Chiou et al., 2019; He et al., 2021), also increased with the evolution of pseudo time, suggesting that M2 macrophages might promote angiogenesis during the progression of LUAD.

Furthermore, we investigated the regulatory activities of transcription factors (TFs) in different trajectory states to understand the regulation of macrophage evolution. Notably, the expression of *KLF3* and *BATF3* was downregulated, whereas the expression of *STAT1* and *TCF4* was upregulated with pseudo time of the trajectory, which might be employed to promote the M2 polarization process (Supplementary Figure S4).

### Proportion of Tregs and plasma B cells increases with the progression of lung adenocarcinoma

**FIGURE 5:**
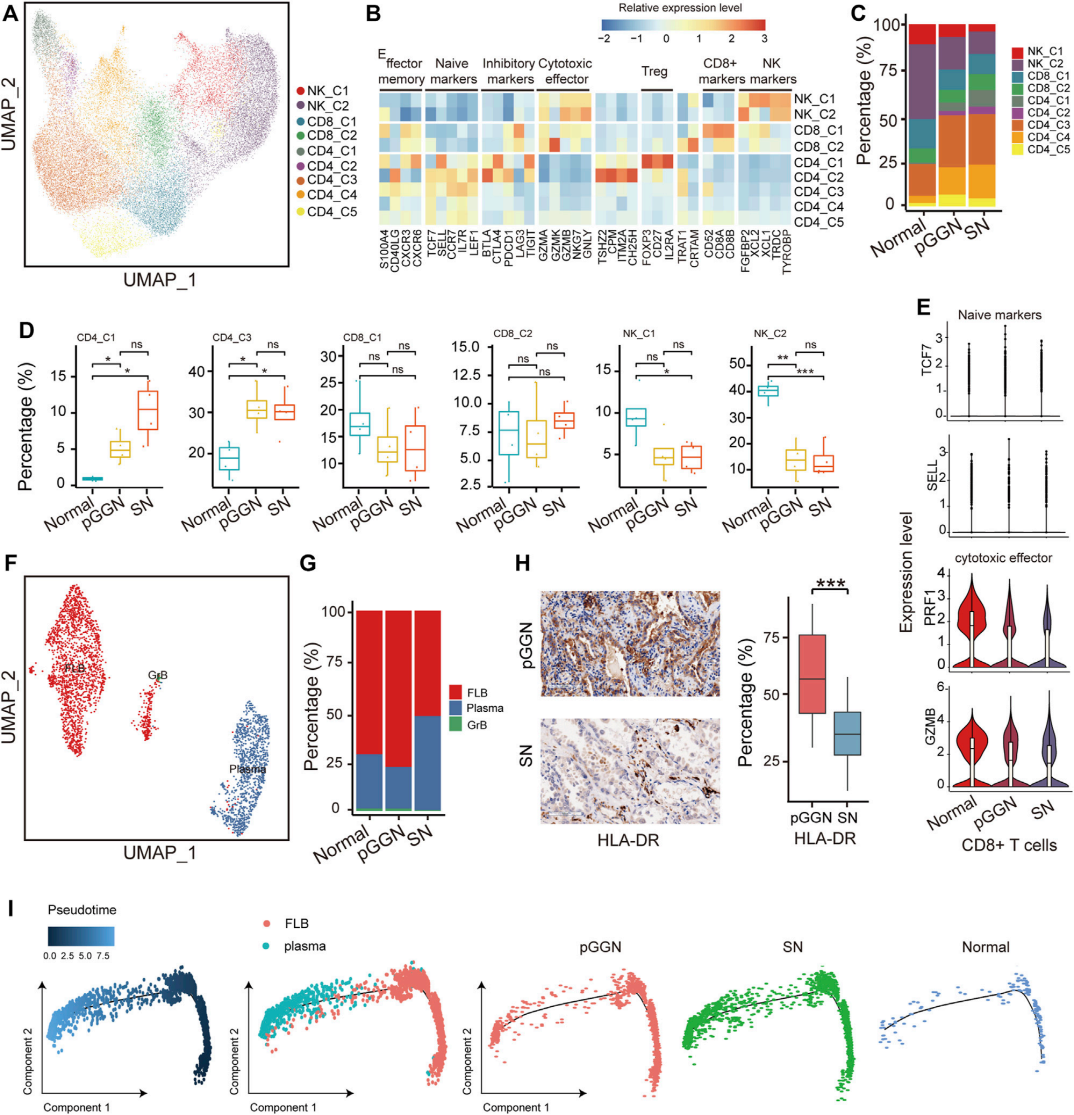
Tregs cells and plasma B cells increased with the progression of LUAD. **(A)** UMAP plot of NK/T cells, colored by 9 cell subsets. **(B)** The heatmap showed the marker genes of NK/T cell subsets. **(C)** The proportion of major cell subsets among each tissue origin. **(D)** Percentages of the cell subsets across pure GGNs, SNs, and normal tissues. Colored dots represent different samples. The comparison was performed using *t*-test. **(E)** The expression distribution of naïve markers and cytotoxic effector markers of CD8^+^ T-cells in each tissue origin. **(F)** UMAP plot of B cells, colored by 3 cell subsets. **(G)** The proportion of cell subsets among each tissue origin. **(H)** The expression levels of HLA-DR in the pure GGN and SN tissues by immunohistochemical examination. The left was the representative images, and the comparison was performed using *t*-test. **(I)** Unsupervised transcriptional trajectory of follicular B cells and plasma B-cells from Monocle2, colored by pseudo time (left), cell types (middle), and tissue origin (right). Ns, *p* > 0.05; *, *p* ≤ 0.05; **, *p* ≤ 0.001; ***, *p* ≤ 0.0001. A *p*-value < 0.05 was considered statistically significant.

With 30,662 cells detected, NK and T cells were the most prevalent cell types. They were subclustered into 9 subsets (Figure 5A) and designated as NK cells (NK-C1 and NK-C2; *TYROBP*+), regulatory T cells (Tregs, CD4-C1; *FOXP3*+), CD4^+^ naïve T cells (CD4-C2, CD4-C3, CD4-C4, CD4-C5; *LEF1*+ and *CCR7*+), and CD8^+^ T cells (CD8-C1, CD8-C2; *CD8B*+ and *CD8A+*) (Figure 5B). Notably, NK cells were depleted from normal tissues to SNs, as previously mentioned (Lavin et al., 2017), while Tregs increased from normal tissues to SNs (Figures 5C,D). Besides, the expression of cytotoxic effector of CD8^+^ T cells decreased in SNs and the expression of naïve markers of CD8^+^ T cells was highly expressed in pure GGNs, suggesting that the cytotoxic function of T cells became weak in SNs (Figure 5E). It is established that Tregs function primarily by repressing the functionality of CD8^+^ T cells in cancer, including LUAD (Ganesan et al., 19502013; Adamczyk et al., 2021). Except for reactivation of CD8^+^ T cells, depleting Tregs may be also therapeutically beneficial.

B cells are also pivotal components of adaptive immunity, except for T cells. Previous studies have also demonstrated that tumor-infiltrating B cells play a critical role in anti-tumor immunity. A total of 3,223 B cells were identified in this study, and they were subclustered into 3 separate subsets based on canonical marker genes: follicular B cells (FLB; *HLA-DRA+*), plasma B cells (*MZB1+*) and granzyme B cells (GrB; *GZMB+*) (Figure 5F; Supplementary Figure S5A). Among these cells, follicular B cells and plasma B cells accounted for most of the total cells. Follicular B cells exhibited high expression of HLA-DRs, and its proportion was relatively higher in pure GGNs than in normal tissues and SNs, indicating that the abundance of follicular B cells increased at an early stage of LUAD (Figure 5G). IHC analysis also supported this result (Figure 5H). Previous studies have certified that follicular B cells are the precursor of plasma B cells, and when they encounter antigen and T-cell help, they can become plasma B cells or memory cells (Pieper et al., 2013). These results imply that follicular B cells may differentiate into plasma B cells with the continuous stimulation of tumor antigens during the progression of LUAD, which leads to the decrease of its abundance.

Plasma B cells are the major resource of antibodies, which are key components of humoral immunity. Of note, the abundance of plasma B cells was relatively higher in SNs (Figure 5G). IHC analysis also revealed that plasma B cells markers like CD138 (Syndecan-1) were upregulated in SNs (Supplementary Figure S5B). These indicated that plasma B cells involved in the progression of LUAD.

In order to further confirm the evolution trajectory of plasma B cells, we extracted follicular B cells and plasma B cells to infer the trajectory and also found that the proportion of plasma B cells from SNs increased in the end of pseudo time (Figure 5I). SCENIC analysis showed that TFs, such as *MYC* and *ELF1*, were depressed, while *XBP1* was activated as pseudo time progressed (Supplementary Figure S5C). Previous studies have implied that *XBP1* may be involved in the development of lymphocytes, including plasma B cells (Manso et al., 2019). We hypothesized that activated *XBP1* and inhibited *MYC* and *ELF1* might promote the differentiation of follicular B cells into plasma B cells.

### Construction of cell–cell interaction networks during the progression of lung adenocarcinoma

To characterize the transformation of intercellular interactions with the progression of LUAD, we, respectively, inferred putative cell-to-cell interactions in pure GGNs and SNs based on ligand-receptor signaling.

**FIGURE 6:**
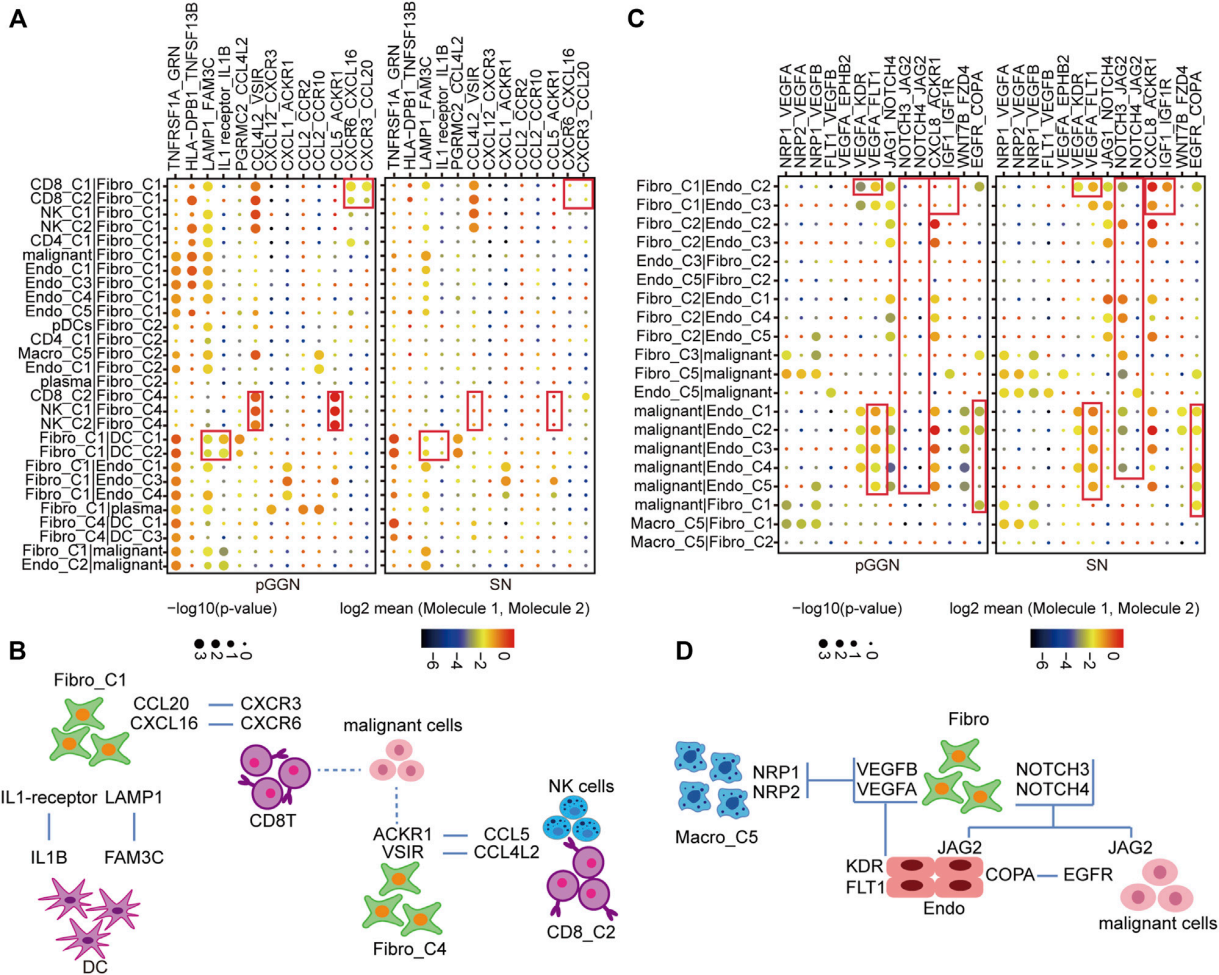
Constructing cell–cell interaction network during the progression of LUAD. **(A)** The dot plot depicting significant interactions of immune response among immune cells and stromal cells from tissues of pure GGN and SN. **(B)** The graphic model of the interactions among immune cells and stromal cell. **(C)** The dot plot depicting significant oncogenic interactions among malignant cells and stromal cells from tissues of pure GGN and SN. **(D)** The graphic model of the oncogenic interactions among stromal cells and malignant cells. The colors of the dot represent the log-transformed expression levels, and the sizes of the bubbles indicate the significance of the interaction, as calculated by CellPhoneDB.

Fibro-C1 could act as an immunosuppressive medium to deliver inflammatory factors and elicit anti-tumor immune response. In the network, CD8 + T cells with high expression of CXCR6 and CXCR3 formed strong cellular communication with Fibro-C1 with high expression of CXCL16 and CCL20. DC interacted with Fibro-C1 in the form of LAMP1_FAM3C and IL1 receiver_IL1B. These interactions were shown to be enhanced in patients with pure GGNs. Moreover, the interactions between CD8^+^ T cells, NK cells, and Fibro-C4 by ligand and receptor binding of CCL4L2_VSIR and CCL5_ACKR1 were more significant in pure GGNs than in SNs (Figures 6A,B).

Furthermore, the interactions that promoted the development of the tumor itself are observed (Figures 6C,D **)**. For instance, VEGF signaling pathway-related interactions, such as VEGFA_KDR and VEGFA_FLT1, were very strong between endothelial cells and fibroblasts. In addition, the NOTCH signaling pathway-related interactions, such as NOTCH3_JAG2 and NOTCH4_JAG2, were detected in malignant cells, endothelial cells, and fibroblasts, which were stronger in SNs than in pure GGNs. Additionally, the EGFR-related interactions were obvious between malignant cells and endothelial cells in SNs. We thought that these intercellular interactions were essential for the development of LUAD.

Meanwhile, we noted that a subset of genes was highly expressed in specific cell subtypes at specific stages. In pure GGNs, LAMP1 and FAM3C were highly expressed in Fibro-C1 and DC-C1, respectively; CCL4L2 and CCL5 were highly expressed in CD8-C2, and VSIR and ACKR1 were highly expressed in Fibro-C4. In SNs, some genes, such as VEGFA, NOTCH3/4, and EGFR, were highly expressed in multiple cell subtypes, which were consistent with our previous findings (Supplementary Figures S6A,B).

We generated a complex interaction network from different origins, including angiogenesis and immune response. The diverse interactions at different development stages of LUAD highlight potential therapeutic targets.

## Discussion

A robust increase of GGNs detected during lung cancer screening program raises the blaring sirens in battle with the most notorious malignancy in the world. Interestingly, these small indolent lesions demonstrate a long history of development into aggressive lesions with systematic spreading potency. The indications for surgical resection are obscure in small pure GGNs, yet routine annual monitoring of these nodules with CT years by years causes considerable psychological stress in patients and significant consumption of medical resources (Zhang et al., 2020). Moreover, synchronous multiple nodules pose a substantial challenge in decision-making during surgery. Hence, there is an urgent need to better understand molecular mechanisms underlying the progression of these small lesions to more invasive entities. In the present study, we depicted the cellular and molecular landscape and dynamics of LUAD from pure GGN to SN using scRNA-seq technology. This study also highlighted different cellular and molecular networks in pure GGNs and SNs, which could facilitate further discovery of therapeutic targets.

Along with progression of LUAD in radiology, we discovered that epithelial cells harbor more malignant characteristics, including the accumulation of CNV and the ability to proliferate. Additionally, the antigen-presenting ability mediated by the MHC molecules is weakened. Usually, the intrinsic characteristics of malignant cells determine tumor biology (Li et al., 2018; Wang et al., 2018). However, efforts to understand the tumor microenvironment have revealed its pivotal role in tumorigenesis (Casey et al., 2015). We found that during the progression of LUAD, innate immunity mediated by NK cells was gradually weakened, and adaptive immunity exhibited significant changes, including the development of T-cell exhaustion and the differentiation from follicular B cells to plasma B cells. Notably, immunosuppression that was mainly depending on myofibroblasts, Tregs, and macrophages was enhanced during this process, and similarly, pro-angiogenesis mediated by M2 macrophages and tECs was also enhanced. Previous studies have also demonstrated that stage I LUADs already harbor significantly altered T-cell and NK-cell components, which support our conclusions. Altogether, the progression of LUAD involves the dynamic reprogramming of multiple cellular elements, including cancer cells, immune cells, and stromal cells. The proliferation of malignant epithelial cells is weak, and immunosurveillance functions well in the early stages. These may together account for the indolent nature of pure GGNs.

T cells are a subpopulation of the most abundant lymphocytes in LUAD and are the main components of adaptive immunity (Guo et al., 2018). They have been widely studied and therapeutically targeted for immunotherapy in various solid tumors. Similarly, B cells are also key components in adaptive immunity with diverse functions. Unfortunately, there are few studies on B cells in LUADs, and inconsistent effects of B cells on tumor development have been reported (Budczies et al., 2021; Cui et al., 2021). B cells mainly produce antibodies and induce T-cell activation and proliferation through antigen presentation to regulate immune and inflammatory response. One previous study revealed that the depletion of B cells *via* anti-IgM antibodies could reduce the tumor burden in mice with colorectal cancer, suggesting a role of B cells in promoting the progression of colorectal cancer (House and Maley, 1986). Another study demonstrated that B cells could enhance the metastatic ability of breast cancer cells by activating the CXCR4/SDF1α axis in tumor cells *via* secreting HSPA4-targeting immunoglobins (Gu et al., 2019). Conversely, several studies have revealed that B cells potentially exhibited anti-tumor functions (Helmink et al., 2020). For instance, depletion of B cells through anti-CD20 antibodies could enhance the progression of melanoma in mice. One recent study based on scRNA-seq provided multiple novel insights of B cells in non-small cell lung cancer, and identified several subtypes of tumor-infiltrating B cells with versatile functions (Chen et al., 2020). In our study, we identified five separate subsets of B cells and found that the abundance of follicular B cells was relatively higher in pure GGNs, while that of plasma B cells was relatively higher in SNs. We thought that this may be due to the differentiation from follicular B cells to plasma B cells with the continuous stimulation of tumor antigens during tumor development. Together, our study identifies that except for T cells, B cells, especially plasma B cells, also play pivotal roles in the progression of LUAD.

In recent years, immunotherapy mainly based on immune checkpoint inhibitors (ICIs), such as anti-PD1/PD-L1 and anti-CTLA-4 antibodies, has significantly altered the landscape of cancer treatment, including lung cancer (Borghaei et al., 2015; Billan et al., 2020). Unfortunately, only a minority of unselected patients benefit from immunotherapy. This is mainly due to the significant complexity of the tumor microenvironment of solid tumor. First, at different developmental stages, immune cells that act as determinants vary. In this study, we found that NK cells were more abundant in pure GGNs, but gradually decreased. This indicates that innate immunity mediated by NK cells is still relatively active at an early stage of LUAD. In the clinic, there are more and more synchronous and metachronous multiple early-stage primary LUAD presenting as pure GGNs or mixed GGNs. Although surgery is the optimal treatment strategy for early-stage LUAD, it is very difficult to resect all GGNs within individual patients because of the limitation of surgical technology or the physical condition of the patient. This poses a substantial challenge for clinicians. One recent study reported the effect of postoperative EGFR-TKI treatment on residual GGO lesions after lung cancer resection (Cheng et al., 2021). Although some patients may benefit from this modality, there are several inherent limitations to this strategy, due to drug resistance and inter-tumor heterogeneity in at genetic level. Based on our study, we propose that NK cell-mediated immunotherapy may be an ideal and effective treatment approach for patients with pure GGN who could tolerate surgery and for patients with multiple GGNs. Fortunately, a wide variety of sources of therapeutic NK cells are currently being clinically tested (Myers and Miller, 2021). Additionally, several other stromal cells, such as CAFs and endothelial cells, also present dynamic changes during the progression of LUAD. These immune and stromal cells constitute a complex ecosystem which facilitates the tumor development. Therefore, combination therapy targeting two or more immune cells and stromal cells may be a reasonable strategy for the refractory advanced stage LUADs that have received ICI monotherapy.

There are some limitations in this study. First, the clonality of T cells and B cells is important in the process of tumor development, however, we did not investigate this. Therefore, further studies based on single-cell TCR and BCR sequencing are imperative in the future. Second, mixed GGN is composed of GGN and solid component and is highly heterogeneous. Therefore, we did not analyze mixed GGNs. Third, due to the technical limitations of scRNA-seq, we could not identify the spatial distribution of immune cells and stromal cells in individual tumor, and new technologies of spatial transcriptomics may be helpful to solve this problem. Fourth, due to technological limitations, we could not further confirm the detailed functions of specific cell types, such B cells. Finally, there are not sufficient functional experiments to verify our results. Therefore, further investigations on cellular and molecular biology are still required in the future.

In summary, we comprehensively revealed the cellular and molecular landscape and dynamics of different stages of resectable LUAD. Anti-tumor immunity mediated by NK and CD8+T cells gradually weakened during the progression of LUAD, and humoral immunity mediated by plasma B cells was more active in late stages. Additionally, stromal cells, including fibroblasts and macrophages, also played pivotal roles in this process. Moreover, there were complex cell–cell interactions among these cells, and they constituted a complex ecosystem to determine the development of LUAD. Our study may facilitate our understanding of LUAD and the discovery of novel therapeutic targets.

## Data Availability

The datasets presented in this study can be found in online repositories. The names of the repository/repositories and accession number(s) can be found below: CNCB-NGDC (National Genomics Data Center, China National Center for Bioinformation, https://ngdc.cncb.ac.cn/gsa/) and the accession number is PRJCA006719.
