# Supplementary material for: Single-cell RNA sequencing reveals the cellular and molecular changes that contribute to the progression of lung adenocarcinoma
Source: Front Cell Dev Biol. 2022 Aug 15;10:927300. doi: 10.3389/fcell.2022.927300 (PMC9420948; doi:10.3389/fcell.2022.927300)
Supplement: Supplementary file 1 [file DataSheet1.docx]

## Supplementary figures


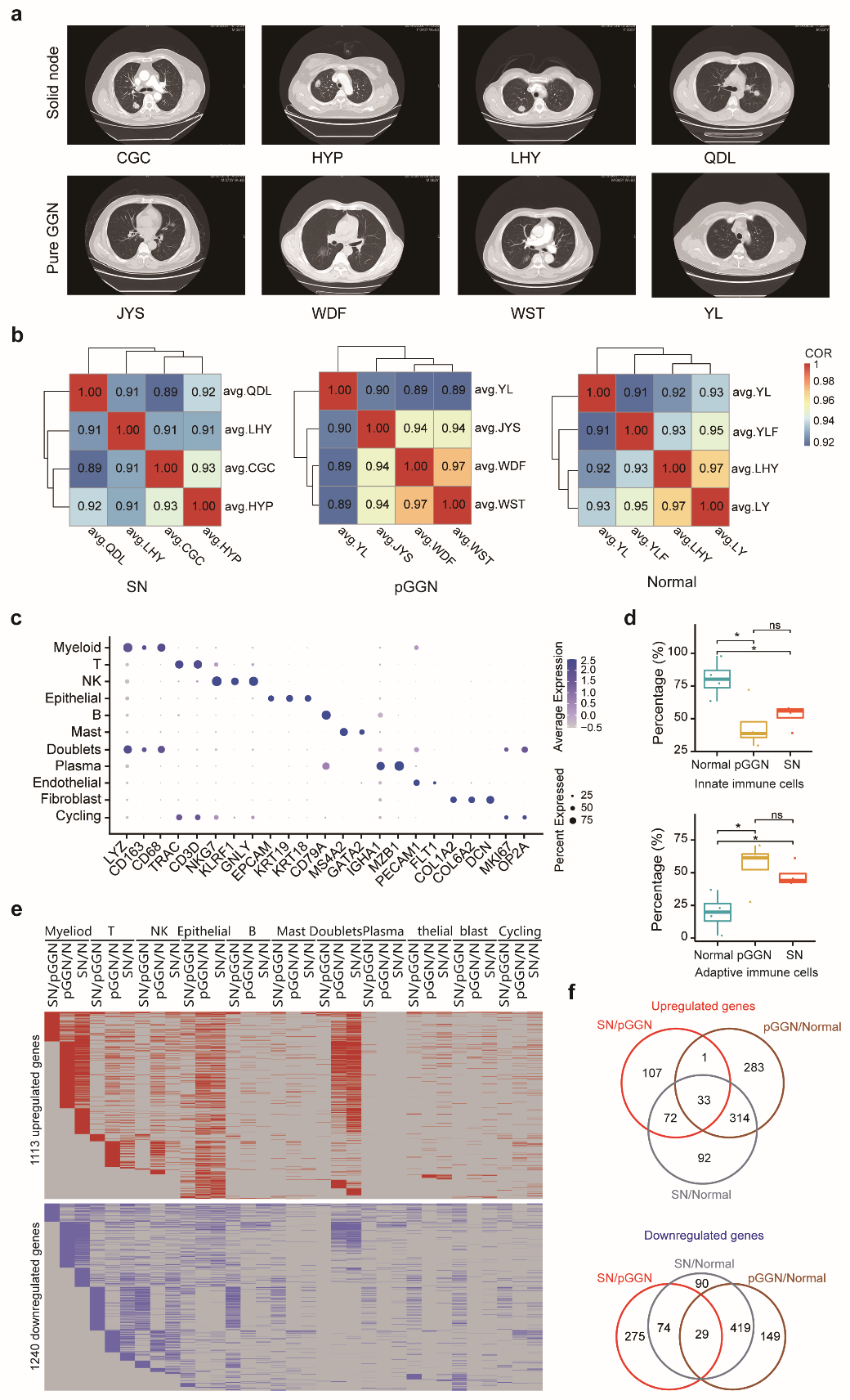


**Supplementary Fig. 1 Single-cell transcriptomic profiling of pure GGN and SN. a** CT images of the patients with SN (upper) and pure GGN (lower) in this study. **b** The average CPM (count per million) of each sample was analyzed by correlation coefficient among the same group of samples. Correlation coefficient was calculated by spearman correlation. **c** Dot plot showed canonical cell type markers. **d** Percentages of the innate immune cells (upper) and adaptive immune cells (lower) among three groups. Colored dots represent different samples. T test was used for significance difference analysis. ns, *P* > 0.05, *, *P* ≤ 0.05, * *, *P* ≤ 0.01, * * *, *P* ≤ 0.001. **e** Heatmaps showing the distribution of upregulated (red) and downregulated (blue) genes for each cell type during the progression of LUAD at the single cell resolution. **f** Venn plots showing the number of upregulated (upper) and downregulated (lower) genes between different groups during the progression of LUAD.


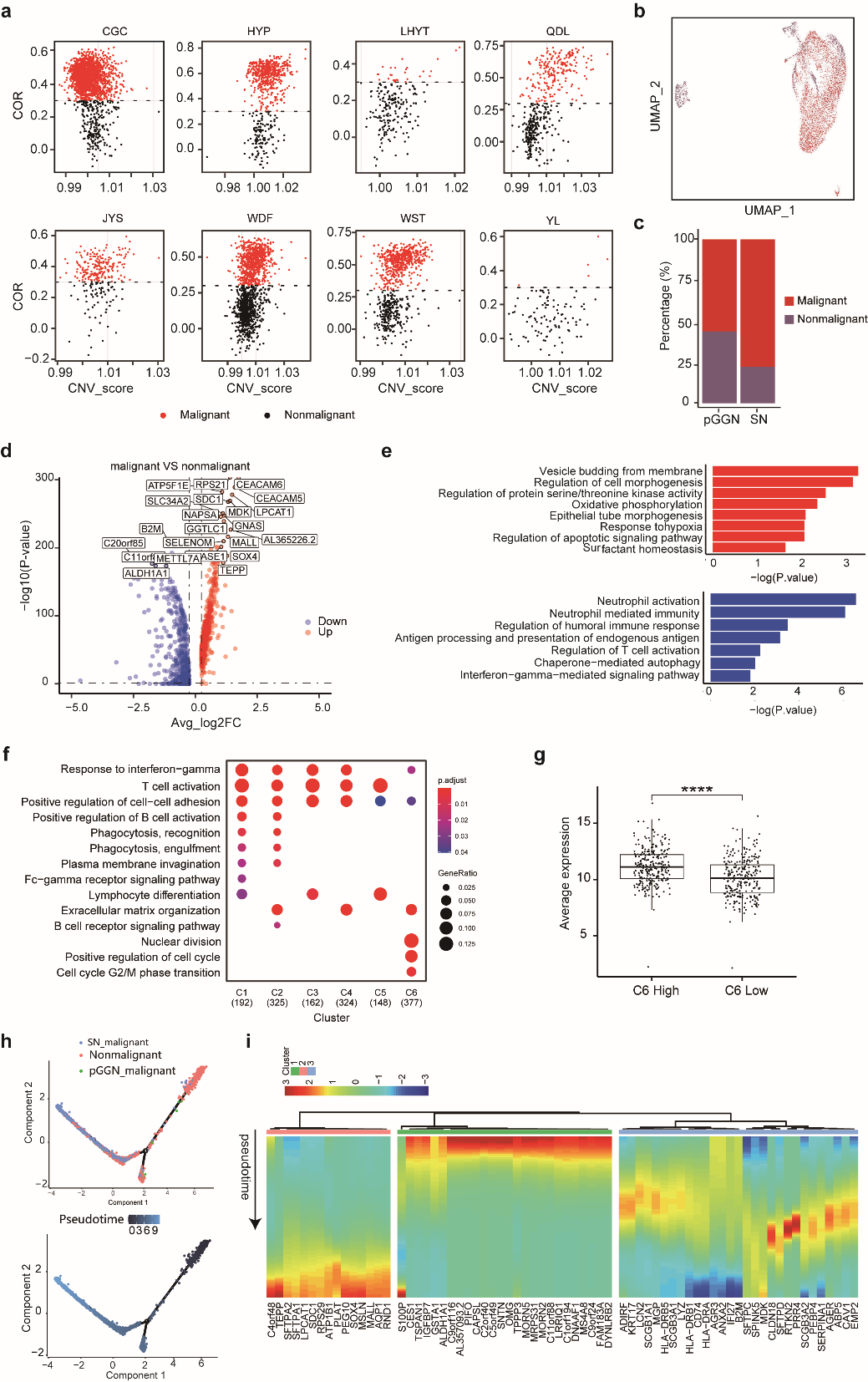


**Supplementary Fig. 2 Epithelial malignant cells had different functions in different tissue origin. a** The dot plot showed the distribution of malignant and non-malignant cells among each sample. X-lab represents CNV score, Y-lab represents correlation coefficient. The horizontal dashed line represented the threshold. **b** UMAP plot of epithelial cells from 8 tumor tissues, colored by malignant cells and nonmalignant cells. The red dots represent malignant cells and the purple ones represent nonmalignant cells. **c** The proportion of major cell types among each tissue origin. **d** Volcano plot of differentially expressed genes between malignant cells and nonmalignant cells from tumors. Upregulated and downregulated genes in malignant cells were colored with red and blue, respectively. **e** The enriched GO terms for upregulated (upper) and downregulated (lower) genes. **f** GO enrichment of genes highly expressed in each cell subclusters. **g** Expression of C6 cluster marker genes in TCGA LUAD patients. According to the expression of C6 cluster marker genes, TCGA LUAD patients were divided into two group, C6-high and C6-low. The p-value was calculated by t-test. ***, *P* ≤ 0.0001. **h** Unsupervised transcriptional trajectory of malignant and nonmalignant cells from Monocle2, colored by cells from different origins (upper) and pseudotime (lower). **i** The heatmap showing genes expression changes with pseudo time.


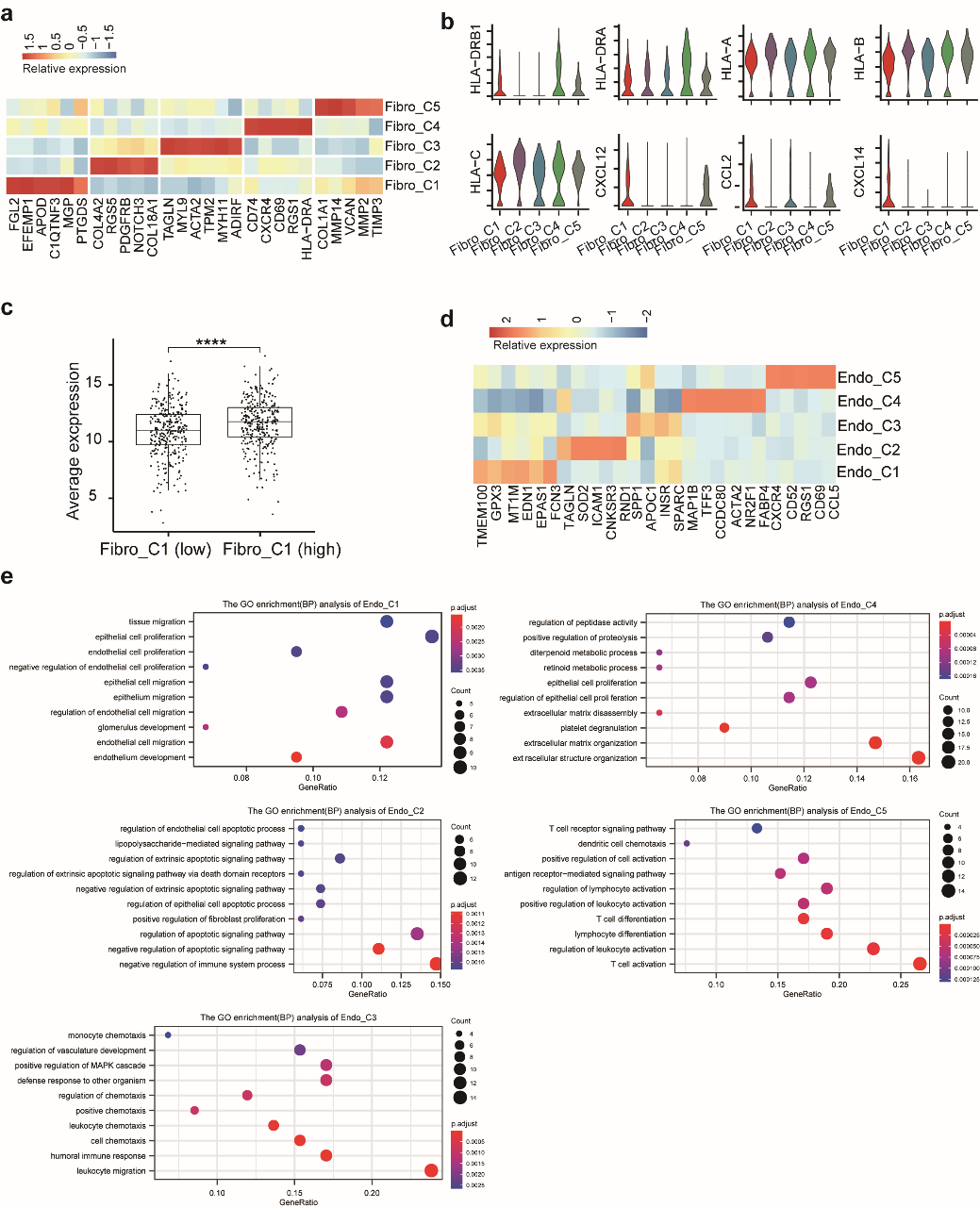


**Supplementary Fig. 3 Stromal cells assist progression of tumor during the progression of LUAD. a** The heatmap showed the marker genes of fibroblast subsets. **b**. Violin plots showed the expression of MHC relative genes and cytokines in fibroblasts subsets. **c** Expression of Fibro-C1 cluster marker genes in TCGA LUAD patients. The p-value was calculated by t-test. ***, *P* ≤ 0.0001. **d.** The heatmap showed the marker genes of endothelial cell subsets. **e.** The dot plot showed top 10 signal pathways by GO analysis in each endothelial cell subsets.


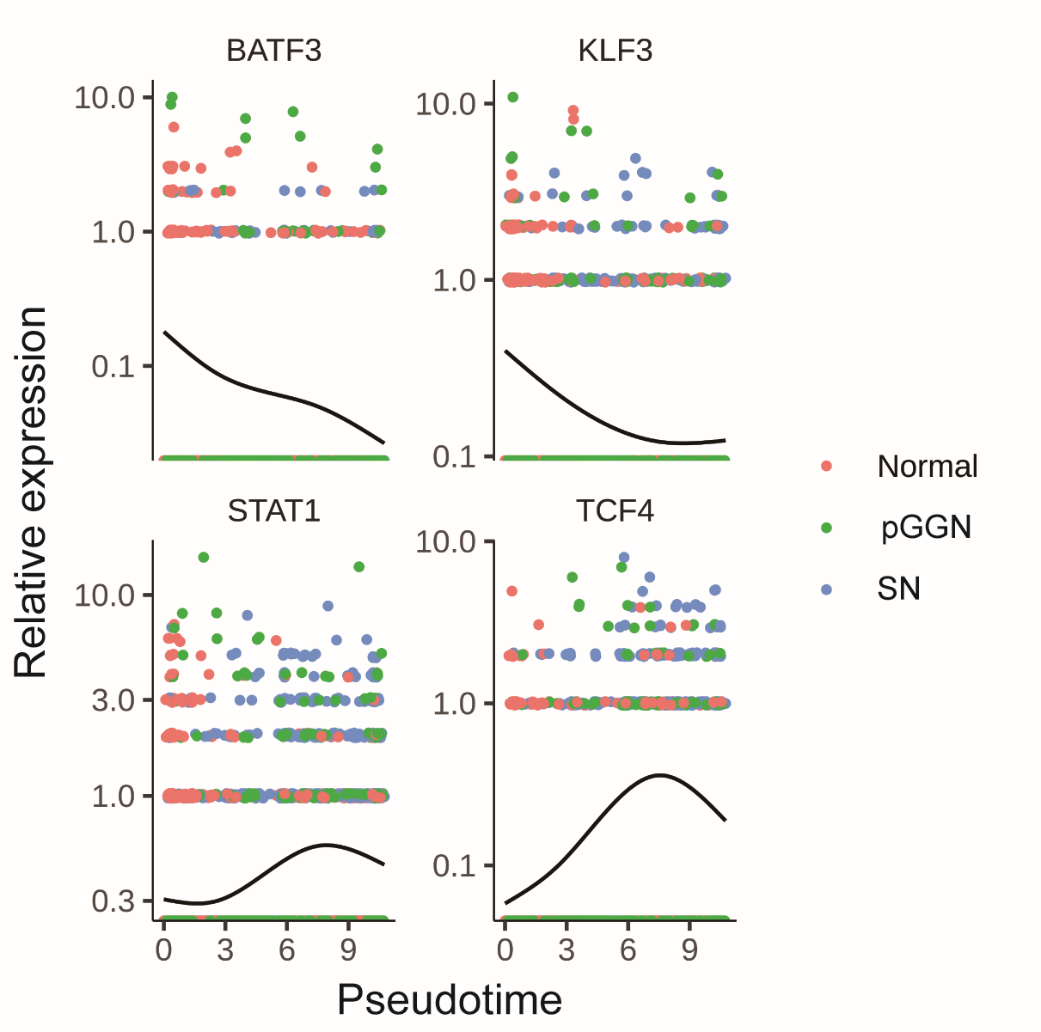


**Supplementary Fig. 4 Pseudo time trajectory of monocytes to macrophages by Monocle2 analysis.** The plot showed changes of expression of transcription factor such as *BATF3*, *KLF3*, *STAT1* and *TCF4* with pseudo time trajectory. Black lines represent the kinetic trends of the genes. The relative expression was normalized by raw counts using the *Size_Factor* function (details in methods). The smooth black line represents the kinetic patterns along the pseudo time trajectory, which is modeled by monocle2. Pure GGNs, SNs and normal tissues are labeled using different colors.


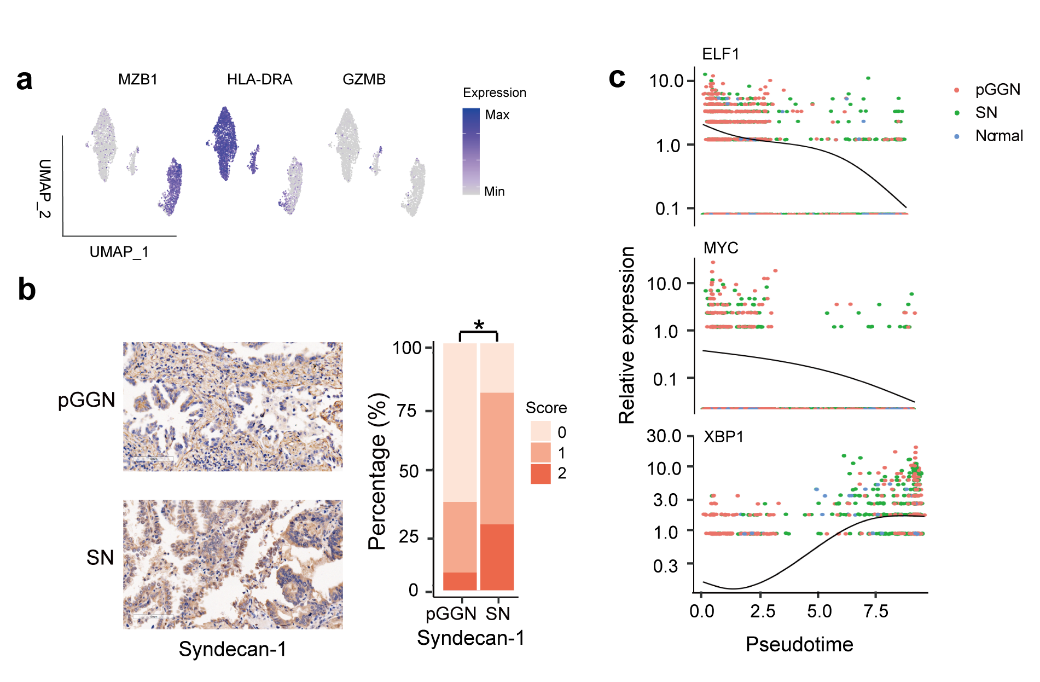


**Supplementary Fig. 5 Plasma B cells increased with the progression of LUAD. a** UMAP plot of the markers of different B cell subsets, colored by gene expression. **b** The representative immunohistochemical images of pure GGN (upper panel) and SN (lower panel) for CD138 (Syndecan-1). The p-value was calculated by t-test. *, *P* ≤ 0.05. **c** The plot showed changes of expression of transcription factor such as *ELF1*, *MYC* and *XBP1* with pseudo time trajectory. The relative expression was normalized by raw counts using the *Size_Factor* function (details in methods). The smooth black line represents the kinetic patterns along the pseudo time trajectory, which is modeled by Monocle2. Pure GGNs, SNs and normal tissues are labeled using different colors.


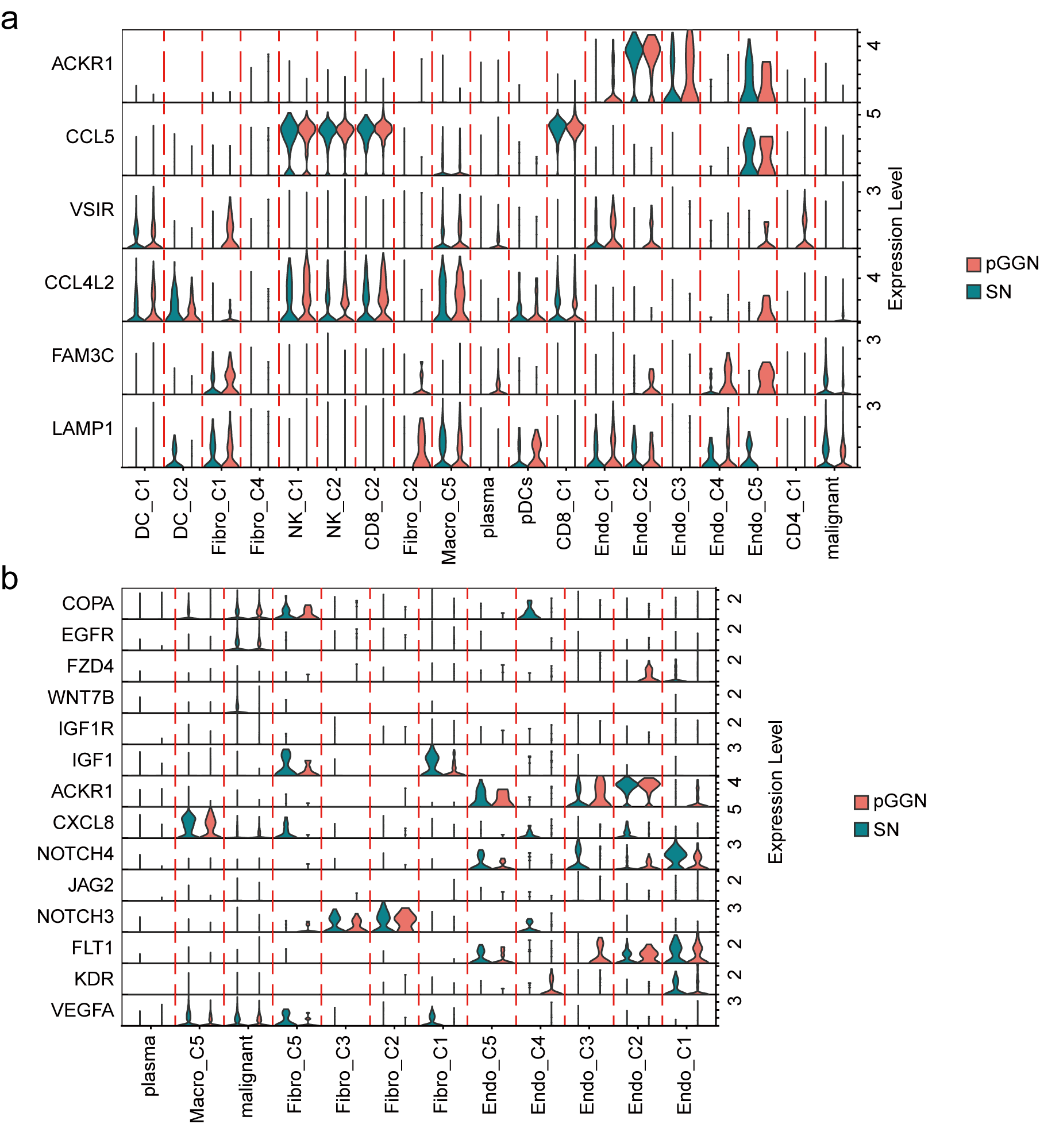


**Supplementary Fig. 6 Expression of receptor and ligand related genes in specific cell subsets from different origins. a** The violin plot depicted the expression of genes associated with immune response among immune cells and stromal cells. **b** The violin plot depicted the expression of genes which were associated with oncogenic signal pathways among malignant cells and stromal cells.
